# Growth and Physiological Responses to Water Depths in *Carex schmidtii* Meinsh

**DOI:** 10.1371/journal.pone.0128176

**Published:** 2015-05-26

**Authors:** Hong Yan, Ruiquan Liu, Zinan Liu, Xue Wang, Wenbo Luo, Lianxi Sheng

**Affiliations:** 1 Key laboratory for Wetland Ecology and Vegetation Restoration of National Environmental Protection, School of Environment, Northeast Normal University, Changchun, China; 2 Jilin provincial academy of forestry science, Changchun, China; 3 Division of Biological Sciences, the University of Montana, Missoula, United States of America; Chinese Academy of Sciences, CHINA

## Abstract

A greenhouse experiment was performed to investigate growth and physiological responses to water depth in completely submerged condition of a wetland plant *Carex schmidtii* Meinsh., one of the dominant species in the Longwan Crater Lake wetlands (China). Growth and physiological responses of *C*. *schmidtii* were investigated by growing under control (non-submerged) and three submerged conditions (5 cm, 15 cm and 25 cm water level). Total biomass was highest in control, intermediate in 5 cm treatment and lowest in the other two submerged treatments. Water depth prominently affected the first-order lateral root to main root mass ratio. Alcohol dehydrogenase (ADH) activity decreased but malondialdehyde (MDA) content increased as water depth increased. The starch contents showed no differences among the various treatments at the end of the experiment. However, soluble sugar contents were highest in control, intermediate in 5 cm and 15 cm treatments and lowest in 25 cm treatment. Our data suggest that submergence depth affected some aspects of growth and physiology of *C*. *schmidtii*, which can reduce anoxia damage not only through maintaining the non-elongation strategy in shoot part but also by adjusting biomass allocation to different root orders rather than adjusting root-shoot biomass allocation.

## Introduction

Water regime is one of the major determinants in plant community dynamics and species zonation in wetlands [1,2]. The water regime can be described by the depth, duration, frequency, rate of filling and drying, and timing and predictability of flooded and dry phases in a wetland [1]. Among these, water depth is considered to be one of the key factors controlling the establishment of wetland species [3]. However, water depth in many wetland systems is not always constant. Current climate models have predicted a much greater frequency of sudden storm events accompanied by rapid water depth increases in lowland wetland habitats [4], which could lead to complete submergence and thus could have severe effects on the growth of wetland plants. Increased water depth can directly increase the time needed for gas exchange to aerate the submerged parts of wetland plants [5,6]. An indirect effect of increased water depth is the attenuation of light in the water column; partial submergence can reduce irradiance to organs and thus reduce carbon assimilation and oxygen production [7]. Therefore, water depth might be one of the key factors in determining wetland species distribution and survival in a completely submerged condition. A number of studies have focused on how wetland plants cope with complete submergence [8–12]. Submergence is detrimental for most plants because it hampers growth and can result in premature death [8]. The negative impact on submerged plants is closely related to the low diffusion rates of gases and the relatively low solubility of oxygen in water [13], which jointly lead to anoxia or hypoxia in plant tissues [14].

Many wetland plants can reduce damage from oxygen deficiency and enhance their ability to tolerate flooding through morphological adjustments [15], such as biomass reallocation [16,17] or adjusting their shoot morphology [5,18]. During floods, some wetland plants can allocate more biomass to aboveground to acquire-oxygen [19], and less biomass to belowground parts in order to reduce oxygen depletion [20]. Changes in shoot morphology are another important strategy in wetland plants when partially or completely submerged [18]. For example, some wetland species can elongate shoot organs such as internodes and petioles during flooding [21], which can help plants bring leaves closer to the surface into better illuminated water layers, and eventually above the water surface. Generally, there are two opposing types of shoot strategies to cope with flooding: shoot elongation (the low-oxygen escape) and non-shoot elongation (the quiescence strategy) [22]. It is suggested that the non-elongation strategy is more advantageous for temporary or deep-flooding events that cannot be outgrown, whereas fast underwater elongation increases fitness mainly during prolonged, but relatively shallow floods [23,24]. Include fast elongation of the shoot is an essential attribute of flooding tolerance in wetland species, which keeps leaves above water in response to rising water level [11,25]. However, increased cell division rates and synthesis of new cell walls during fast cell elongation require substantial amounts of energy and carbohydrates [26], and this strategy is beneficial only if the contact between leaf blades and the atmosphere is restored. Thus, fast elongation under water will be a high-risk strategy during flooding events of short duration and when submergence depths exceed the elongation capacity of the plant [18]. Therefore, submergence depths might be one of the key factors in determining which strategies species adopt in submerged conditions. However, few papers have focused on the responses of wetland species to water depth in submerged conditions [27].

Wetland plants under the flooding conditions can also reduce anoxia damage through physiological adjustments [28]. For instance, enhancing alcohol dehydrogenase (ADH) activity can alleviate damage in plant tissues from oxygen deficiency by maintaining carbohydrate metabolism [29]. Malondialdehyde (MDA) content, a marker for lipid peroxidation, is often used as an indicator of cellular membrane damage [30].

All mechanisms are important for wetland plants to meet the energy requirement of some crucial physiological activities under anoxic conditions [31], so that the starch stored in plants as the primary reserve carbohydrate can be transformed into soluble sugar, such as glucose and fructose, to be used for further metabolism [31]. Thus, accumulation of carbohydrates prior to flooding in plant tissues may also be important for plant survival in flooding environments.

The aim of this study was to identify the role of water depth in affecting the growth and physiology of a wetland plant. For this purpose, *Carex schmidtii* Meinsh. was chosen as the target species for growth in three completely submerged conditions, quantified by water depths of 5 cm, 15 cm and 25 cm for 28 days. *C*. *schmidtii* (a perennial grass, with typical adventitious root system) is one of the dominant species in wet meadows and marsh communities in the Longwan Crater Lake wetlands, the largest Crater Lake marsh in China.

Here, we tested the following hypotheses: First, water depth might have negative effects on plant growth when the plant is completely submerged, and more biomass will be allocated to shoots and less to roots in submerged treatments. Second, plants will adopt the non-elongation strategy in 25 cm submergence conditions, which is advantageous for deep-flooding events that cannot be outgrown. Third, the plant will store more starch at 25 cm submergence, as it is important for wetland species to maintain metabolism when submerged.

## Materials and methods

### PLANT materials

Ramets of *C*. *schmidtii* were chosen in October 2009, from Longwan Freshwater Marsh Field Observation Station (N 42°20', E 126°21'), of Northeast Normal University. We also collected soil from the *C*. *schmidtii* population in the Longwan Freshwater Marsh Field Observation Station. The major soil types are peats. All plants and soil were taken back to a greenhouse at Northeast Normal University, where the temperature was controlled at 25 ± 2°C in the day and 17 ± 2°C at night and light was provided by 400 watt sodium lamps (Guangming Company, China) at a photon flux density of 400/0 μmol m^-2^ s^-1^ (PAR) in a 14 h light/10 h dark cycle. Plant cuttings were then placed into plastic buckets containing 10 cm soil (500 g) and 2 cm water, to germinate new ramets.

### Experimental treatment

**Fig 1:**
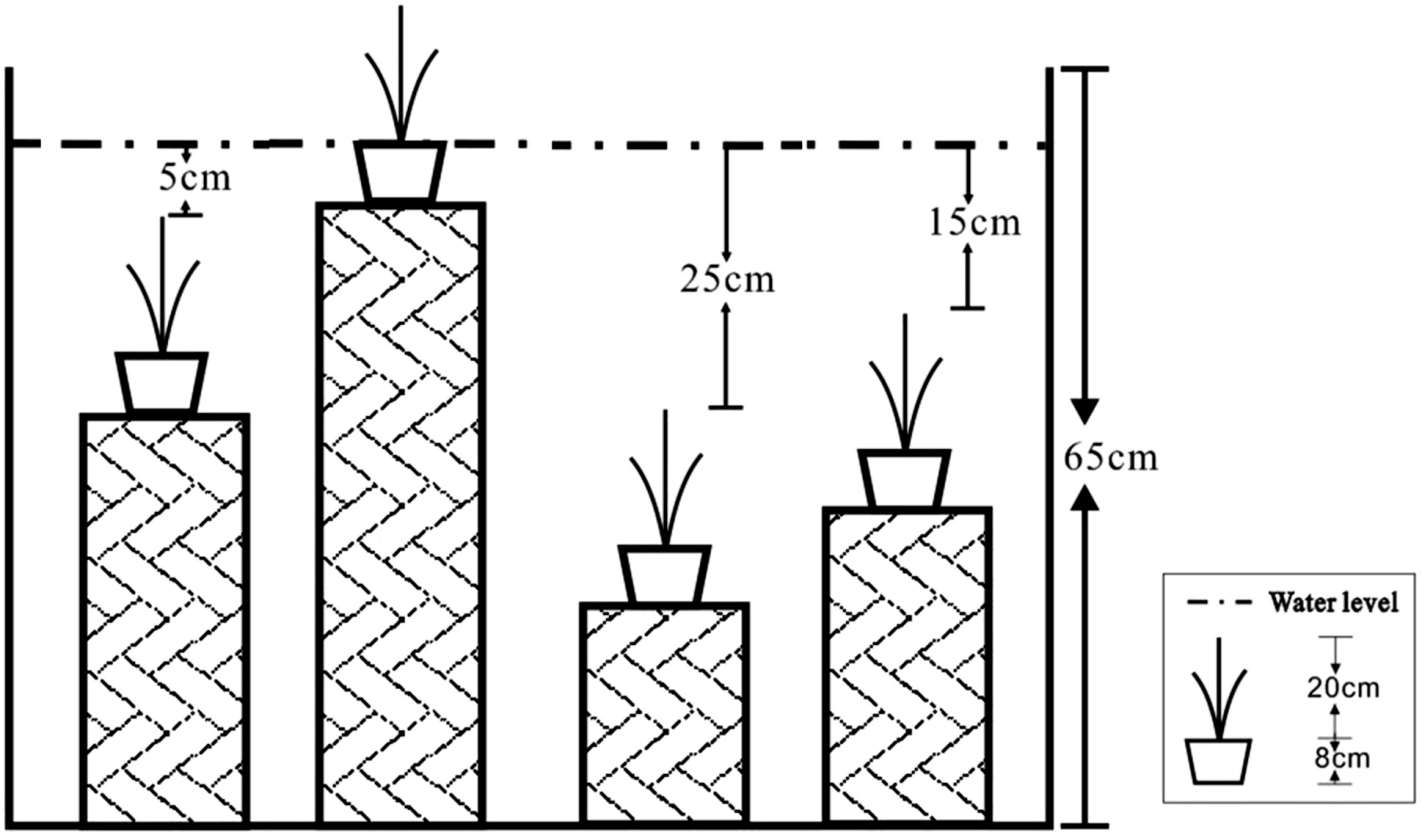
The schematic diagram of the experimental set-up.

A total of 36 plants of similar size (3–4 leaves, about 20 cm in height) were cut from plant cuttings and planted in plastic pots (8 cm in height and 10 cm in diameter, one plant per pot). Each pot was filled with soil, a mixture of 0–30 cm surface soil collected from the same site where the *C*. *schmidtii* population was located (N 42°20', E 126°21'). Four pots were placed into one larger plastic bucket (300L, 65 cm in height) to control water level (three pots per water level). Nine plastic buckets were used in the experiment and were randomly placed in the greenhouse. Every week these plastics buckets were moved randomly. Considering the field observations, we chose 0 cm, 5 cm, 15 cm and 25 cm water levels as experimental flooding levels. The four water levels relative to the soil surface were 0 cm (control), 25 cm (5 cm treatment, completely submerged 5 cm), 35 cm (15 cm treatment, completely submerged 15cm) and 45 cm (25 cm treatment, completely submerged 25 cm) (Fig 1). Tap water was supplied daily to maintain water level.

### Harvest

For each treatment, 9 plants were harvested after 28 days. Before harvest, we recorded the height of each plant (natural height), and after harvest, all plants were divided into shoots and roots to determine fresh weight, respectively. Adventitious roots in each group were then divided into main roots and, first-order laterals (no plants had second-order laterals in submerged conditions at the end of the experiment) and were weighed separately [17]. Half of the shoot and root mass was weighed, oven dried at 80°C for 48 h, and then weighed again to calculate a wet-to-dry conversion factor for each tissue type, which was used for calculated the dry weights (S1 File). After that, the dried tissues were used to determine the carbohydrate content. Another half of the samples were kept in a refrigerator prior to being analyzed for ADH activity, and MDA content [28]. Carbohydrate content, ADH activity and Malondialdehyde (MDA) were measured following the procedure of Wignarajah et al. [32], Cakmak and Horst [33] and Yoshida et al. [34], respectively (S1 File).

### Total biomass and partitioning

Total biomass was calculated using the follow formula: Total biomass = Shoot mass + Root mass. Relative growth rate (RGR) was calculated using the follow formula: RGR = [ln (w_2_)—ln (w_1_)] / (t_2_-t_1_) [35,36], where w_2_ and w_1_ are plant dry weights at the end of the experiment (t_2_) and plant dry weight at the beginning of the experiment (t_1_), respectively. Relative shoot growth rate (RSR) was calculated using the follow formula: RSR = [ln (h_2_)–ln (h_1_)] / (t_2_-t_1_), where h_2_ and h_1_ are plant heights at the end of the experiment and at the beginning of the experiment, respectively.

### Statistical analysis

All statistical analyses were performed using SPSS 20.0 software (SPSS Inc., USA). Treatment effects on total biomass, shoot mass, root mass, root to shoot ratio, first-order laterals to main roots ratio, relative growth rate (RGR), relative shoot growth rate (RSR), ADH activity, MDA content, and carbohydrate content were tested by the analysis of one-way ANOVA. Multiple comparisons of means were performed by Duncan’s test at the 0.05 significance level. Heterogeneity was tested using Levene’s test and data were log10-transformed if necessary to reduce the heterogeneity of variances.

## Results and Discussion

### Relationship between total biomass, shoot mass, root mass, and water level

**Fig 2:**
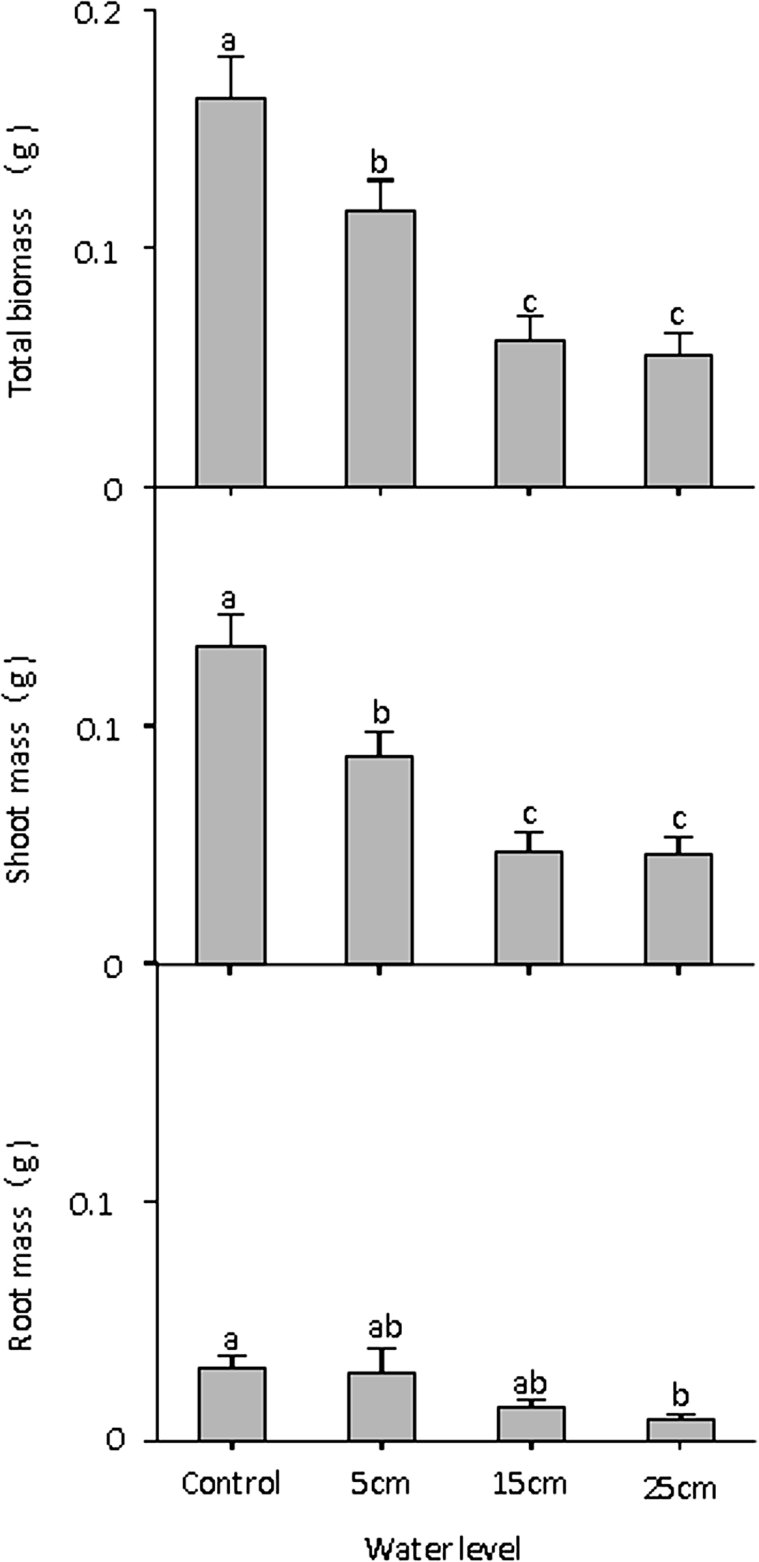
Total biomass, shoot mass, and root mass of *Carex schmidtii* in different treatments (one control: 0 cm water level, and three submerged conditions: 5 cm, 15 cm, and 25 cm water level). Different letters indicate significant differences among treatments (*P* < 0.05).

Water level had significant effects on total biomass and shoot mass, but had no significant effects on root mass and the root to shoot ratio (Table 1). Total biomass and shoot mass decreased significantly with increasing water depth (Fig 2). At end of the experiment, the total biomass were highest in control (0.16 g), intermediate in 5 cm submergence level (0.12 g), and lowest in other submergence levels (0.06 g in 15 cm treatment and 0.05 g in 25 cm treatment). The total biomass and RGR in our experiment showed that water depth might have negative effects on plant growth under the condition of complete submergence, a result that partly supported our first hypothesis. In particular, optimal partitioning models and theories suggested that plants can respond to resource availability by adjusting biomass allocation patterns to optimize resource capture (e.g. nutrients, light) in a manner that maximizes plant growth [37]. Oxygen is one of the limiting resources in wetland ecosystems. Wetland species could reallocate biomass patterns when flooding, allocating more biomass to shoot parts in order to get more oxygen and allocating less biomass to root parts in order to reduce oxygen depletion [19,20]. Therefore, adjustment in biomass allocation is also an important strategy to enable wetland plants to survive during flooding periods [16].

**Table 1:** Summary of one-way ANOVAs for total biomass, ADH activity, and carbohydrates contents of *Carex schmidtii* in different water-level conditions.

	n	Treatment (F- statistics)	Treatment (P-values)
Total biomass (g)	9	18.223^***^	0.000
Shoot mass (g)	9	19.007^***^	0.000
Root mass (g)	9	2.888^ns^	0.510
Root to shoot ratio (%)	9	2.838^ns^	0.530
First-order laterals to main roots ratio (%)	9	4.184^*^	0.013
Relative growth rate (d^-1^)	9	18.424^***^	0.000
Relative shoot growth rate (d ^-1^)	9	62.696^***^	0.000
ADH activity (μ g^-1^ fw)	5	7.707^**^	0.003
MDA content (μ mol g^-1^)	5	4.486^*^	0.018
Starch content (mg g^-1^)	3	0.633 ^ns^	0.617
Soluble sugar content (mg g^-1^)	3	41.400^***^	0.000

^ns^ *P* > 0.05

^*^ *P* < 0.05

^**^ *P* < 0.01

^***^ *P* < 0.001

### Relationship between first-order laterals to main roots ratio and water level

**Fig 3:**
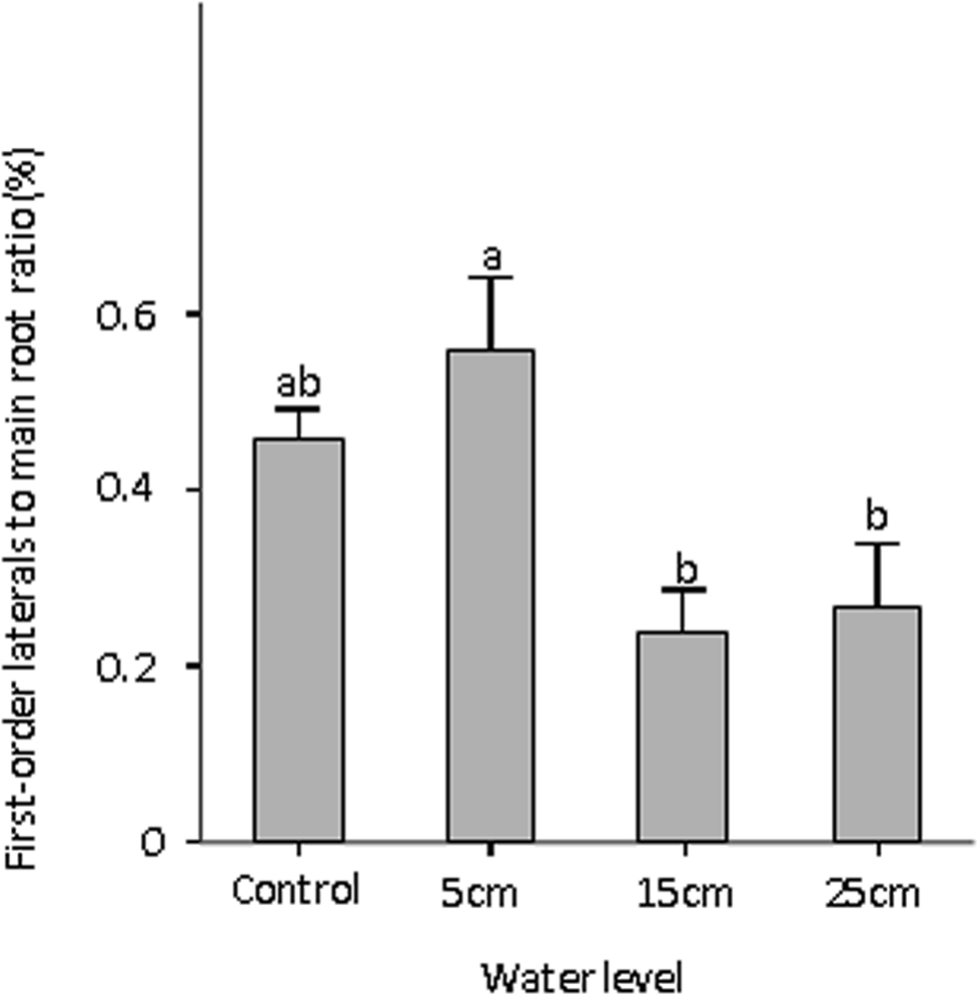
First-order laterals to main roots ratio (%) of *Carex schmidtii* in different treatments (one control: 0 cm water level, and three submerged conditions: 5 cm, 15 cm, and 25 cm water level). Different letters indicate significant differences among treatments (*P* < 0.05).

The first-order laterals to main roots ratio showed the same trend as total biomass, indicating a significant effect of water depth (Table 1). At the end of the experiment, the first-order laterals to main roots ratios were higher in control and 5 cm treatment than the other treatments (Fig 3). However, root to shoot ratio was not altered in our experiment, which partly contradicted our first hypothesis that more biomass will be allocated to shoots and less to roots in submerged treatments. However, an interesting finding was that *C*. *schmidtii* can allocate less biomass to first-order laterals as water depth increase. Xie et al. reported that the wetland plant *Myriophyllum spicatum* could reduce the damage from anoxia by adjusting root structure and biomass allocation to different root orders rather than through root morphology [17]. During submerged conditions, the decreased biomass allocation to lateral roots could also reduce the total root numbers or root lengths, which in turn could reduce radial oxygen loss or reduce oxygen depletion by root system, so a low mass fraction of lateral roots is favorable for survival in submerged environments. Therefore, these data indicated that reallocating biomass to different root orders rather than shoot parts might be a strategy used by *C*. *schmidtii* when completely submerged.

### Relationship between relative growth rate, relative shoot growth rate, and water level

**Fig 4:**
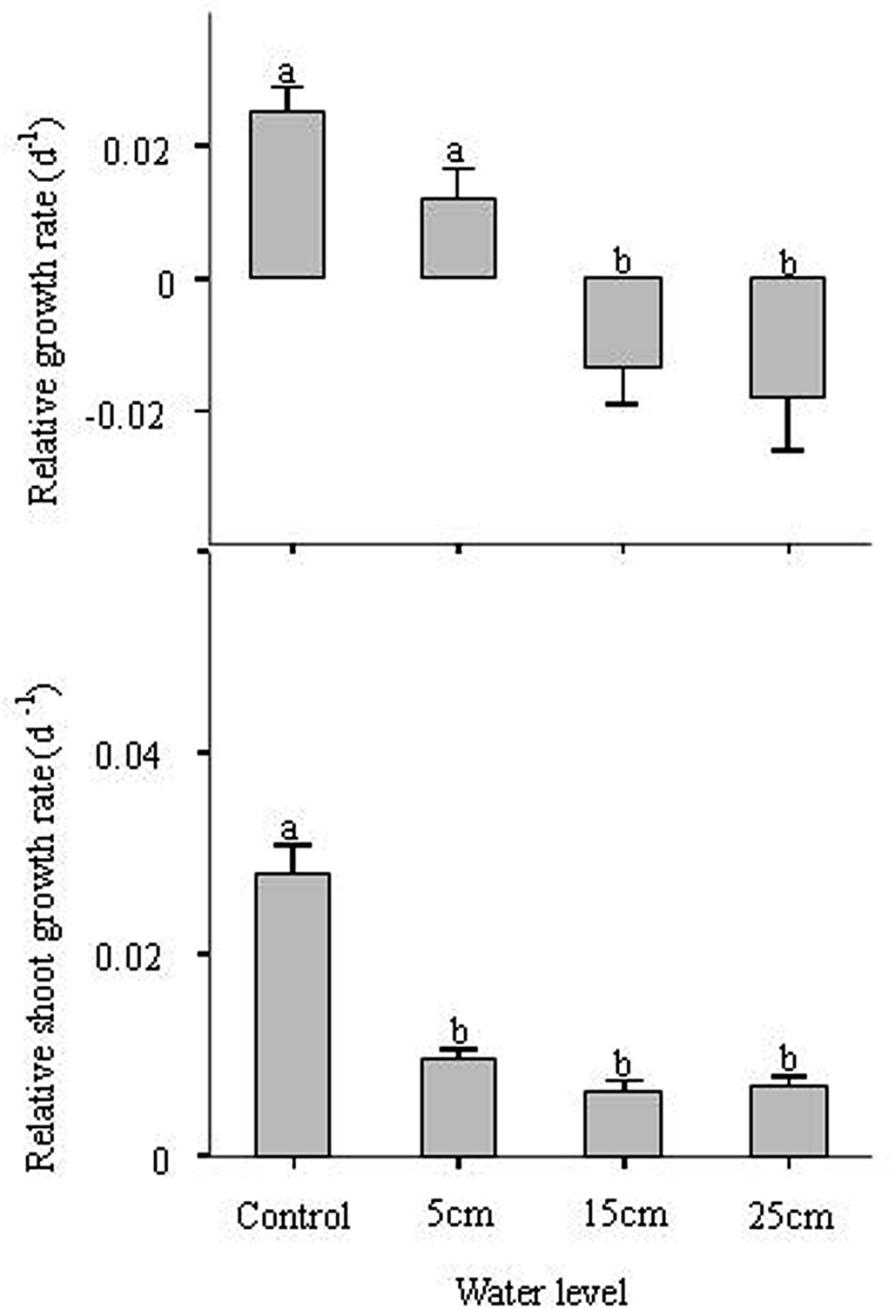
Relative growth rate and relative shoot growth rate of *Carex schmidtii* in different treatments (one control: 0 cm water level, and three submerged conditions: 5 cm, 15 cm, and 25 cm water level). Different letters indicate significant differences among treatments (*P* < 0.05).

Relative growth rate (RGR) was higher in control and 5 cm treatments but lower in others (Table 1, Fig 4). The relative shoot growth rate (RSR) was higher in control than the other treatments for *C*. *schmidtii* (Fig 4). Rapid stem elongation occurs mainly to minimize reduction of photosynthesis, which concomitantly increases O_2_ production, which can be internally transported by aerenchyma to submerged tissues in an otherwise anoxic environment. However, in our experiment, RSR was lower in submerged treatments than that in control. This result indicates that the stems of *C*. *schmidtii* did not elongate in all submerged conditions and submergence might be stressful for plant growth, which confirmed our second hypothesis that *C*. *schmidtii* would adopt non-elongation strategy in 25 cm submerged conditions. Therefore, we can find a positive relationship between RGR and RSR in *C*. *schmidtii* (Fig 4). They were all higher in control but lower in 15 cm and 25 cm treatments (Fig 4). Shoot elongation underwater requires energy and carbohydrates for cell divisions as well as the synthesis of new cell-wall material [5,23]. Therefore, reduced energy consumption in submerged environment might be a survival strategy for *C*. *schmidtii*, which confirmed the hypothesis that the non-elongation strategy might be more advantageous for temporary or deep-flooding events that cannot be outgrown [23,24]. However, the mechanisms by which the plants determine whether the water depth can be overcome or not still need further study.

### Relationship between ADH, MDA, and water level

**Fig 5:**
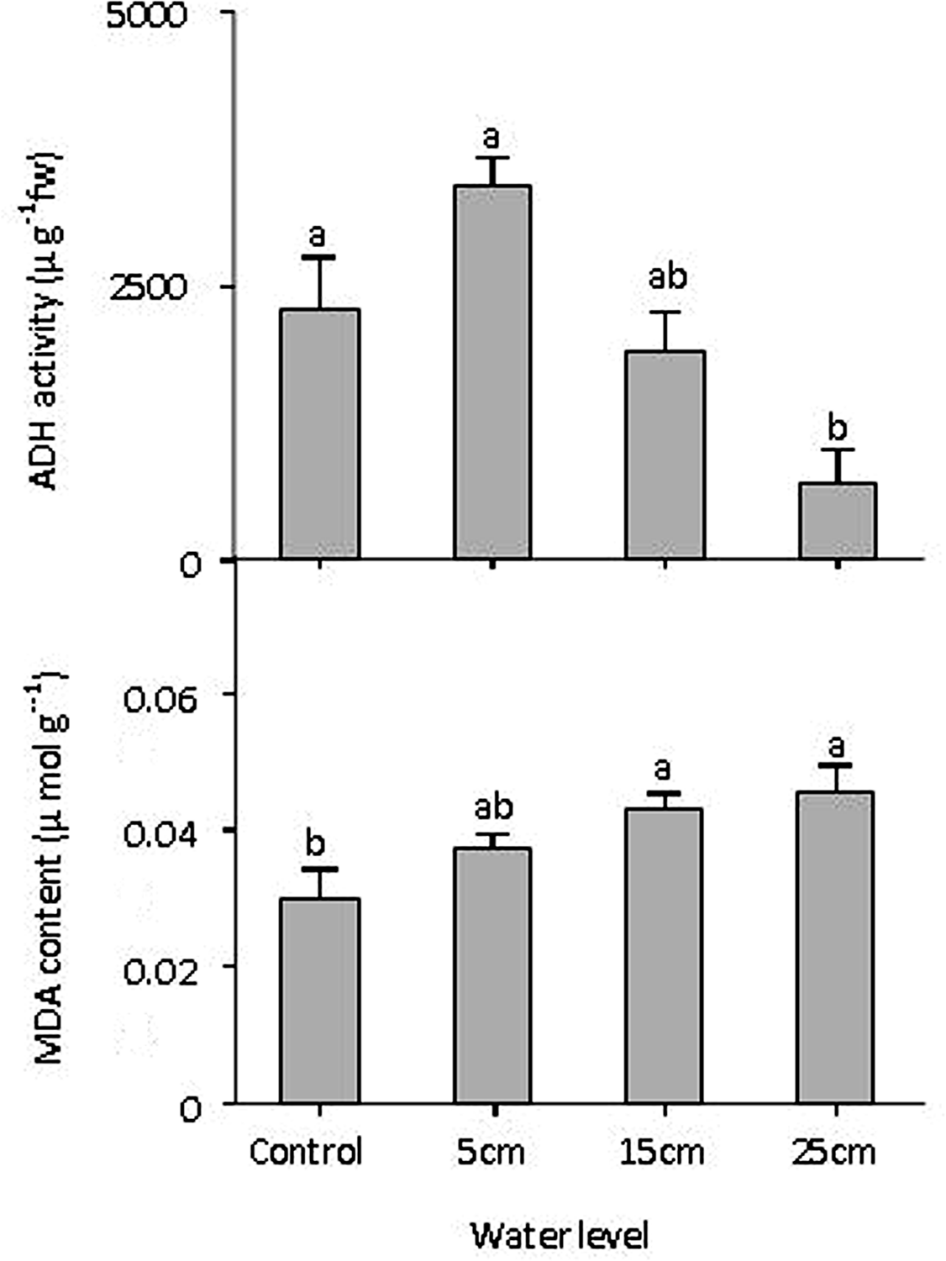
ADH activity and MDA content of *Carex schmidtii* in different treatments (one control: 0 cm water level, and three submerged conditions: 5 cm, 15 cm, and 25 cm water level). Different letters indicate significant differences among treatments (*P* < 0.05).

ADH activity decreased with increasing water depth (Table 1, Fig 5). At the end of the experiment, ADH activity was higher in control and 5 cm treatments and lower in 15 cm and 25 cm treatments (Fig 5). MDA content was significantly and positively affected by water depth (Table 1, Fig 5). In general, ADH activity is usually enhanced when the oxygen supply to roots is limited [17], allowing wetland plants to survive flooding conditions. However, ADH in *C*. *schmidtii* decreased as water depth increased. MDA content in plant tissues can indicate the degree of damage caused by severe stress [38,39]. The higher MDA content in 15 cm and 25 cm treatments suggested that water depth had particularly severe impacts on plant growth in submerged conditions. More importantly, aerobic metabolism in submerged or flooding conditions enables plants to tolerate oxygen deficiency at the cellular level [40,41]. Flooding can lead to a switch of aerobic metabolism into less efficient anaerobic fermentation, causing a fast depletion of carbohydrate reserves [8]. In other words, pre-stored non-structural carbohydrates (including starch and soluble sugar) are important for wetland plant survival in anoxic environments [42].

### Relationship between starch content, soluble sugar content, and water level

**Fig 6:**
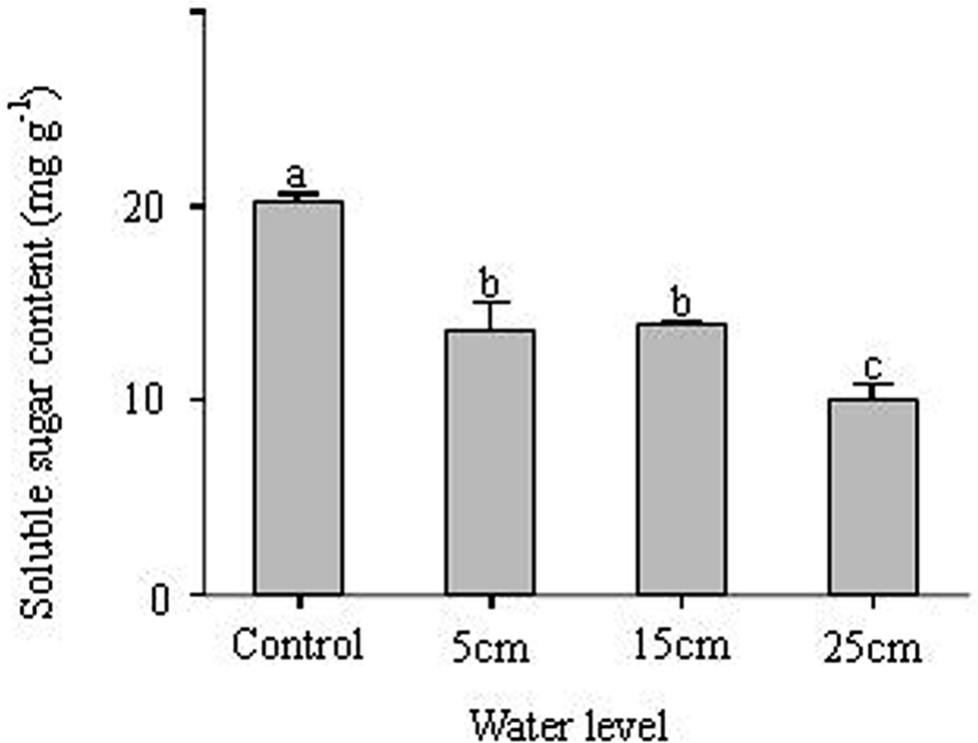
Soluble contents of *Carex schmidtii* in different treatments (one control: 0 cm water level, and three submerged conditions: 5 cm, 15 cm. and 25 cm water level). Different letters indicate significant differences among treatments (*P* < 0.05).

Water level did not have significant effects on starch content (Table 1). However, soluble sugar content was significantly and negatively affected by water level (Table 1, Fig 6). There were no differences in starch content among different submerged treatments in our experiment, which contradicted our third hypothesis that *C*. *schmidtii* might store more starch in the 25 cm submerged treatment. Therefore, it seems that *C*. *schmidtii* might have stored enough starch to maintain metabolism when submerged. But under anoxic conditions, many more carbohydrates were depleted to satisfy the energy requirement of crucial physiological activity [43,44], so starch must be transformed into soluble sugar for normal anoxic metabolism [31]. This could indicate that the ability to transform starch to soluble sugar might be crucial for plant survival in anoxic conditions. However, in our experiment the lower soluble sugar content in the 25 cm submerged condition suggested that *C*. *schmidtii* might not be capable of producing enough soluble sugar for anoxic metabolism. In summary, our experiment indicates that submergence depth might affect total biomass and anoxic metabolism, but adaptations, such as the non-elongation strategy in the shoot part or adjustments in the root structures, allow *C*. *schmidtii* to survive complete submergence.

## Conclusions

Total biomass was highest in control, intermediate in 5 cm treatment, and lowest in the other two submerged treatments. Water depth prominently affected the first-order lateral root so as to main root mass ratio. Alcohol dehydrogenase (ADH) activity decreased but malondialdehyde (MDA) content increased as water depth increased. The starch contents showed no differences among different treatments at the end of the experiment. However, soluble sugar contents were highest in control, intermediate in 5 cm and 15 cm treatments, and lowest in 25 cm treatment. Our data suggest that submergence depth affected some aspects of growth and physiology of *C*. *schmidtii*, which can reduce anoxia damage not only through maintaining the non-elongation strategy in shoot part but also by adjusting biomass allocation to different root orders rather than adjusting root-shoot biomass allocation.

## Supporting Information

S1 File Original data. (XLSX)

## References

[1] Casanova MT, Brock MA. How do depth, duration and frequency of flooding influence the establishment of wetland plant communities? Plant Ecol; 2000: 147: 237–250.

[2] Barrat-Segretain M-H, Cellot B. Response of invasive macrophyte species to drawdown: The case of *Elodea* sp. Aquat Bot. 2007; 87: 255–261.

[3] Budelsky RA, Galatowitsch SM. Effects of water regime and competition on the establishment of a native sedge in restored wetlands. J Appl Ecol. 2000; 37: 971–985.

[4] Zedler JB. How frequent storms affect wetland vegetation: a preview of climate-change impacts. Frontiers in Ecology and the Environment. 2010; 8: 540–547.

[5] Voesenek LACJ, Colmer TD, Pierik R, Millenaar FF, Peeters AJM. How plants cope with complete submergence. New Phytol. 2006; 170: 213–226. 16608449 10.1111/j.1469-8137.2006.01692.x

[6] Rich SM, Pedersen O, Ludwig M, Colmer TD. Shoot atmospheric contact is of little importance to aeration of deeper portions of the wetland plant *Meionectes brownii*; submerged organs mainly acquire O_2_ from the water column or produce it endogenously in underwater photosynthesis. Plant Cell Environ. 2013; 36: 213–223. 10.1111/j.1365-3040.2012.02568.x 22734500

[7] Edwards AL, Lee DW, Richards JH. Responses to a fluctuating environment: effects of water depth on growth and biomass allocation in *Eleocharis cellulosa* Torr. (Cyperaceae). Can J Bot. 2003; 81: 964–975.

[8] Bailey-Serres J, Voesenek LACJ. Flooding stress: acclimations and genetic diversity. Annu Rev Plant Biol. 2008; 59: 313–339. 10.1146/annurev.arplant.59.032607.092752 18444902

[9] Colmer TD, Pedersen O. Underwater photosynthesis and respiration in leaves of submerged wetland plants: gas films improve CO_2_ and O_2_ exchange. New Phytol. 2008; 177: 918–926. 18086222 10.1111/j.1469-8137.2007.02318.x

[10] Fukao T, Harris T, Bailey-Serres J. Evolutionary analysis of the Sub1 gene cluster that confers submergence tolerance to domesticated rice. Ann Bot-London. 2009; 103: 143–150. 10.1093/aob/mcn172 18824474 PMC2707309

[11] Pierik R, van Aken JM, Voesenek LACJ. Is elongation-induced leaf emergence beneficial for submerged *Rumex* species? Ann Bot-London. 2009; 103: 353–357. 10.1093/aob/mcn143 18697756 PMC2707306

[12] Rich SM, Ludwig M, Colmer TD. Aquatic adventitious root development in partially and completely submerged wetland plants *Cotula coronopifolia* and *Meionectes brownii* . Ann Bot-London. 2012; 110: 405–414. 10.1093/aob/mcs051 22419759 PMC3394642

[13] Jackson MB. Ethylene-promoted Elongation: an Adaptation to Submergence Stress. Ann Bot-London. 2008; 101: 229–248. 17956854 10.1093/aob/mcm237 PMC2711016

[14] Armstrong W, Drew MC. Root growth and metabolism under oxygen deficiency In: Waisel Y, Eshel A, Kafkafi U. editors. Plant roots: the hidden half, New York, NY, USA: Marcel Dekker: 2000 pp. 729–761.

[15] Fraser LH, Karnezis JP. A comparative assessment of seeding survival and biomass accumulation for fourteen wetland plant species grown under minor water-depth differences. Wetlands. 2005; 25: 520–530.

[16] Blom CWPM, Voesenek LACJ. Flooding: the survival strategies of plants. Trends Ecol Evol. 1996; 11: 290–295. 21237846 10.1016/0169-5347(96)10034-3

[17] Xie YH, Luo WB, Ren B, Li F. Morphological and physiological responses to sediment type and light availability in roots of the submerged plant *Myriophyllum spicatum* . Ann Bot-London. 2007; 100: 1517–1523. 17959731 10.1093/aob/mcm236 PMC2759222

[18] Ridge I. Ethylene and growth control in amphibious plants In: Crawford RMM, editors. Plant life in aquatic and amphibious habitats. Blackwell Scientific Publications, Oxford, UK;1987 pp. 53–76.

[19] Chen X, Visser EJW, De Kroon H, Pierik R, Voesenek LACJ, Huber H. Fitness consequences of natural variation in flooding induced shoot elongation in *Rumex palustris* . New Phytol. 2011; 190: 409–420. 10.1111/j.1469-8137.2010.03639.x 21261627

[20] Visser EJW, Bögemann GM, Van de Streeg HM, Pierik R, Blom CWPM. Flooding tolerance of *Carex* species in relation to field distribution and aerenchyma formation. New Phytol.2000; 148: 93–103. 10.1046/j.1469-8137.2000.00742.x 33863031

[21] Chen X, Pierik R, Peeters AJM, Poorter H, Visser EJW, Huber H, et al Endogenous ABA as a key switch for natural variation in flooding-induced shoot elongation. Plant Physiol. 2010; 154: 969–977. 10.1104/pp.110.162792 20699400 PMC2949041

[22] Bailey-Serres J, Voesenek LACJ. Life in the balance: a signaling network controlling survival of flooding. Curr Opin Plant Biol; 2010: 13: 489–494. 10.1016/j.pbi.2010.08.002 20813578

[23] Setter TL, Laureles EV. The beneficial effect of reduced elongation growth on submergence tolerance of rice. J Exp Bot. 1996; 47: 1551–1559.

[24] Sauter M. Rice in deep water: “How to take heed against a sea of troubles”. Naturwissenschaften, 2000; 87: 289–303. 11013876 10.1007/s001140050725

[25] Colmer TD, Voesenek LAC. Flooding tolerance: suites of plant traits in variable environments. Funct Plant Biol. 2009; 36: 665–681. 10.1071/FP09144 32688679

[26] Voesenek LACJ, Rijnders JHGM, Peeters AJM, van de Steeg HM, de Kroon H. Plant hormones regulate fast shoot elongation under water: from genes to communities. Ecology. 2004; 85: 16–27.

[27] Wang QL, Chen JR, Liu F, Li W. Morphological changes and resource allocation of *Zizania latifolia* (Griseb.) Stapf in response to different submergence depth and duration. Flora. 2014; 209: 279–284.

[28] Luo WB, Song FB, Xie YH. Trade-off between tolerance to drought and tolerance to flooding in three wetland plants. Wetlands. 2008; 28: 866–873.

[29] Albrecht BG, Biemelt S, Baumgartner S. Accumulation of fructants following oxygen deficiency stress in related plant species with different flooding tolerances. New Phytol. 1997; 136: 137–144.

[30] Sofo A, Dichio B, Xiloyannis C, Masia A. Lipoxygenase activity and proline accumulation in leaves and roots of olive trees in response to drought stress. Physiol Plantarum. 2004; 121: 58–65. 15086818 10.1111/j.0031-9317.2004.00294.x

[31] Li YZ, Xie YH, Ren B, Luo WB, Huang JS. Oxygen enhances the recovery of *Potamogenton maackianus* from prolonged exposure to very low irradiance. Aquat Bot. 2007; 86: 295–299.

[32] Wignarajah K, Greenway H, John CD. Effect of waterlogging on growth and activity of alcohol dehydrogenase in Barley and Rice. New Phytol. 1976; 77: 585–592.

[33] Cakmak I, Horst WJ. Effect of aluminum on lipid peroxidation, superoxide dismutase, catalase, and peroxidase activities in root tips of soybean (Glycine max). Physiol Plantarum. 1991; 83: 463–468.

[34] Yoshida S, Forno DA, Cock JH. Laboratory manual for physiological studies of rice. IRRI, Los Baños, Philippines, 1971.

[35] Xie YH, An SQ, Wu BF. Resource allocation in the submerged plant *Vallisneria natans* related to sediment type, rather than water-column nutrients. Freshwater Biol. 2005; 50: 391–402.

[36] Luo WB, Xie YH. Growth and morphological responses to water level and nutrient supply in three emergent macrophyte species. Hydrobiologia. 2009; 624: 151–160.

[37] McConnaughay KDM, Coleman JS. Biomass allocation in plants: ontogeny or optimality? A test along three resource gradients. Ecology. 1999; 80: 2581–2593.

[38] Hernandez JA, Almansa MS. Short-term effects of salt stress on antioxidant systems and leaf water relations of leaves. Physiol Plantarum. 2002; 115: 251–257. 12060243 10.1034/j.1399-3054.2002.1150211.x

[39] Munné-Bosch S, Alegre L. Plant aging increases oxidative stress in chloroplasts. Planta, 2002; 214: 608–615. 11925044 10.1007/s004250100646

[40] Crawford RMM, Brandle R. Oxygen deprivation stress in a changing environment. J Exp Bot. 1996; 47: 145–159.

[41] Nabben RHM, Blom CWPM, Voesenek LACJ. Resistance to complete submergence in *Rumex* species with different life histories: the influence of plant size and light. New Phytol. 1999; 144: 313–321.

[42] Das KK, Sarkar RK, Ismail AM. Elongation ability and nonstructural carbohydrate levels in relation to submergence tolerance in rice. Plant Sci. 2005; 168: 131–136.

[43] Webb T, Armstrong W. The effects of anoxia and carbohydrates on the growth and viability of rice, pea and pumpkin roots. J Exp Bot. 1983; 34: 579–603.

[44] Alcoverro T, Zimmerman RC, Kohrs DG, Alberte RS. Resource allocation and sucrose mobilization in light-limited eelgrass *Zostera marina* . Mar Ecol Prog Ser. 1999; 187: 121–131.

